# Instant Transport Media for Biopsied Soft Tissue Specimens: A Comparative Study

**DOI:** 10.1155/2015/876531

**Published:** 2015-02-22

**Authors:** Shankargouda Patil, Roopa S. Rao, Anveeta Agarwal, A. Thirumal Raj

**Affiliations:** Department of Oral Pathology & Microbiology, Faculty of Dental Sciences, M.S. Ramaiah University of Applied Sciences, Bangalore 560054, India

## Abstract

*Background.* Formalin, a traditionally preferred fixative in the field of pathology, has restricted usage in private settings. Since its toxicity violates the Occupational Safety and Health Administration regulations, an eco-friendly alternative would be the need of the hour. Hence an instant media which is economical and nontoxic and enables easy transport of biopsied soft tissue specimens in its original state is of vital importance. *Materials and Methods.* Commercially available fresh goat buccal mucosa specimens were sliced into smaller bits of equal dimensions and placed in six different containers containing 20% honey, 30% jaggery, milk, and ice for 1 hr, 6 hours, 12 hours, and 24 hours each with formalin as a positive control. After the set time interval, the specimens were fixed in 10% buffered formalin for 24 hours followed by routine processing and staining. The histologic interpretations were a blinded procedure and evaluated by two experts. Results were statistically analysed. *Results.* 30% jaggery proved to be an ideal transport media showing high quality preservation after 24 hours. 20% honey and ice showed optimal tissue preservation up to 6 hours following which quality deteriorated. Tissues transported in milk showed poor preservation. *Conclusion.* 30% jaggery can be endorsed in routine histopathological analysis as a transport media.

## 1. Introduction

It has been over a century since Ferdinand Blum discovered the ability of formalin to fix tissues, thereby preserving them to be examined later. Since then formalin has been exploited in the field of histopathology as the ideal fixative. Due to its high potency and cost effectiveness, it has been an adorned fixative [1, 2]. Though formalin is economical, its toxic nature and need for storage in a sealed compartment have prompted private practitioners to look for transport media to preserve the tissue until it can be transported to a laboratory for fixation and subsequent processing. Further the toxic nature of formalin is well documented by a number of international agencies (European Union, International Agency for Research on Cancer, and World Health Organization) [3, 4]. The present study was not an attempt to replace formalin as a fixative, but an endeavour to preserve the soft tissue specimens temporarily in various available natural products like honey [5–10], jiggery [5, 11, 12], milk [13–17], and ice before fixing them in 10% buffered formalin solution. Honey and jaggery are eco-friendly, economical, and their ease of availability makes them a prime alternative to formalin.

In the present study routine haematoxylin and eosin (H and E) staining was carried out to assess the diagnostic value of the tissues after placement in the selected transport media for various set time intervals and they were compared to the tissue directly placed in formalin.

## 2. Materials and Methods

**Figure 1:**
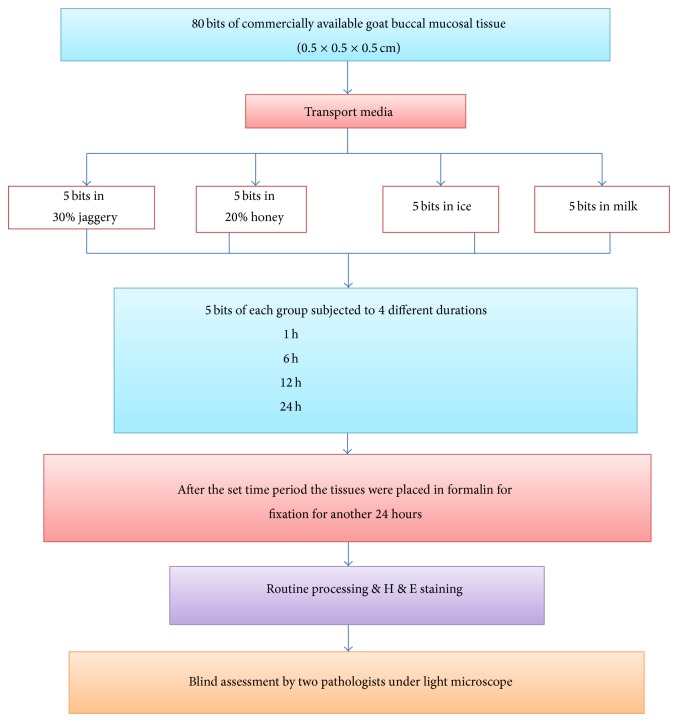
Flowchart representing the protocol employed in the study.

The study was done to assess and compare the quality of the biopsied soft tissues preserved in various transport media for set time intervals (1 hour (hr), 6 hrs, 12 hrs, and 24 hrs) with formalin as a positive control. Commercially available fresh goat buccal mucosa specimens (*n* = 80) were cut into smaller bits measuring approximately 0.5 × 0.5 × 0.5 cm each. 5 tissue bits were placed in six different containers each containing 10% buffered formalin, distilled water, 20% honey, 30% jaggery, milk, and ice for specific amounts of time intervals, namely 1 hr, 6 hrs, 12 hrs, and 24 hrs. Formalin was taken as the positive control and distilled water as negative control. The solutions were at room temperature, except milk which had to be stored in the refrigerator at 5 degrees Celsius. After the set time periods the tissues were removed from the respective transport media and fixed in 10% buffered formalin for another 24 hrs, following which standard processing and H&E staining were undertaken.  Figure 1 summarises the entire procedure. The histologic sections were assessed for histomorphological criteria enlisted in Table 1. The criteria were set on a scale of 1–4 (1 being poor and 4 being excellent) adapted from Patil et al. [5]. Two experts were used to assess the histomorphological features and the whole procedure was blinded. The scores were analysed using Kruskal Wallis ANOVA test. Kappa statistics were carried out for inter observer variability.

## 3. Results

**Figure 2:**
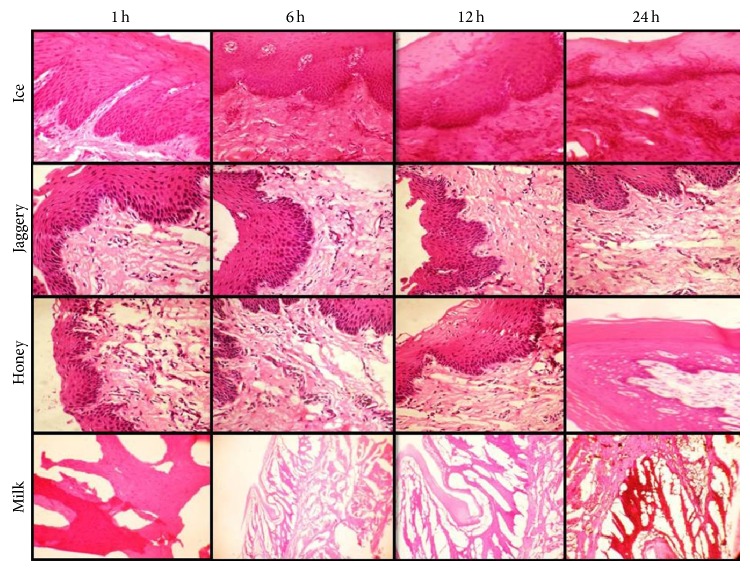
Photomicrograph of the tissues fixed in ice, 30% jaggery, 20% honey, and milk for 1 hr, 6 hrs, 12 hrs, and 24 hrs, respectively (H&E, 10x).

Among the four transport media, tissues preserved in 30% jaggery elicited superior morphology followed by 20% honey and ice. The photomicrographs of H&E stained soft tissue specimens are depicted in Figure 2. The results obtained by Kruskal Wallis ANOVA test were depicted in Table 2.

At 1 hr ice, 20% honey and 30% jaggery showed comparable results with all tissues scored as good or excellent in contrast to milk which showed poor architectural details and all the tissues were categorised as poor or satisfactory.

All the tissue bits placed in 30% jaggery for 6 hrs showed good preservation of detail followed by ice and 20% honey. Milk was again scored the least with all of the tissues being scored as poor or satisfactory.

At 12 hrs, 30% jaggery proved to be superior to other solutions. At 12 hours, tissues placed in ice showed deterioration and loss of morphological details. Though tissue architecture was maintained in all tissues placed in 20% honey for 12 hours, cytoplasmic and nuclear details were satisfactorily appreciated. Tissues preserved in milk were nondiagnostic.

Tissues placed in 30% jaggery for 24 hrs showed good preservation of the overall architecture of the tissue compared to 20% honey, ice, and milk which showed considerable distortion and lack of details.

Statistically significant results were obtained with tissues placed in 30% jaggery even up to a period of 24 hrs (*P* < 0.001). 20% honey preserved tissue sections showed adequate preservation of architecture up to 12 hrs. After 12 hrs, there was a progressive loss of tissue quality denoted by swelling of cells, nuclear shrinkage and loss of cohesiveness. Ice preserved tissue maintained architectural integrity up to 6 hrs, following which cells displayed lysis, fragmentation, and architectural loss. Milk proved to be a poor transport medium and gave disappointing results for a minimum period of an hour. Tissues preserved in milk were difficult to section due to improper impregnation of wax and were shredded.

To collate the findings of the present study, 30% jaggery proved to be the ideal transport media with 20% honey as a successor in line.

## 4. Discussion

Ideally, it is the fixative that decides the fate of the tissue section. Hence freshly biopsied tissue should be fixed immediately in formalin, reflecting adequate preservation of tissue architecture and cytoplasmic details. This is invariably not always possible in a private set-up, possibly due to nonavailability of formalin. The difficulty in the subsequent transport of biopsied tissues in its natural state has prompted a search for an instant transport media.

Numerous studies have been conducted in an attempt to replace formalin. Though few studies have shown significant results, none possessed the quality of formalin fixation. The present study was not an attempt to replace formalin as a fixative, but an endeavour to preserve biopsied soft tissue specimens for varying time durations before attempting fixation.

The concentration selected for honey (20%) and jaggery (30%) was based on the study conducted by Patil et al. The possible mechanism by which jaggery fixes tissues could be attributed to the presence of fructose which at low pH breaks down to aldehydes and cross-links with the amino acids present in the tissue. Being a natural fixative which is readily available and economical, it is required to endorse its use as a universal transport media. The cytoprotective and antioxidant property of jaggery as demonstrated by Nayaka et al. makes it an ideal solution as a transport media [5, 11, 12].

Honey has been used as an antibacterial agent for the treatment of ulcers and bed sores and other surface infections resulting from burns and wounds. In a study conducted by Al-Maaini and Bryant on tissues fixed in honey, the results were found to be comparable to formalin fixed tissue. The present study demonstrated that 20% honey can be a quality preservative up to 6 hrs beyond which deterioration begins. Mechanism of fixation of honey is similar to that of jaggery by means of aldehyde amino acid cross-linking [5–10].

Ice is an established mode of transport in the field of organ transplantation, but its use in preserving biopsy specimen for longer durations is debatable. The present study suggests the use of ice as a transport media for a period of 6 hrs if other means of storage are not available. The tissues should be transferred to formalin within 6 hrs for preservation of diagnostic details.

Blomlöf et al. and Trope and Friedman recommended milk as an excellent storing solution due to the presence of nutritional substances like amino acids, vitamins, and carbohydrates. Milk is indicated as a storage media for avulsed teeth, by the American Association of Endodontics, but it failed to preserve the tissue details and proved to be a poor holding solution for biopsied tissues. Moreover, sectioning tissues placed in milk were cumbersome as wax impregnation was not complete leading to shredded and discontinuous sections [13–17].

30% jaggery showed results comparable to formalin preserved tissues. Ice and 20% honey showed preservation up to 6 hours, beyond which the tissue started losing its architecture. Milk preserved tissues had the most inferior quality. Given the low cost of jaggery in comparison to honey and its relative superiority, we propose 30% jaggery to be a stable transport media for tissue specimens.

## 5. Conclusions

In the present study, jaggery provided optimal tissue preservation comparable to that of formalin fixed tissues. Honey and ice showed moderate to questionable results, while milk proved to be a poor transport medium. Hence, we conclude that jaggery, an eco-friendly alternative, can be endorsed as a routine transport media for biopsied soft tissue specimens.

## Figures and Tables

**Table 1:** Histomorphological criteria.

Criteria	Scale (each criterion was evaluated on a scale of 1–4)
(A) Cellular outline (B) Cytoplasmic detail (C) Nuclear detail (D) Staining quality (E) Overall morphology	(1) Poor (2) Satisfactory (3) Good (4) Excellent

**Table 2:** Summary of the results after evaluation of the tissues placed in various solutions for specific amounts of time (only cases scoring good and excellent were considered).

Duration	Criteria	ICE (*n* = 5)	20% honey (*n* = 5)	30% jaggery (*n* = 5)	Milk (*n* = 5)	*P* value
1 hours	Cellular outline	5 (100%)	5 (100%)	5 (100%)	0 (0%)	<0.001
	Cytological detail	5 (100%)	5 (100%)	5 (100%)	0 (0%)	
	Nuclear detail	5 (100%)	5 (100%)	5 (100%)	0 (0%)	
	Staining quality	5 (100%)	5 (100%)	5 (100%)	0 (0%)	
	Overall morphology	5 (100%)	5 (100%)	5 (100%)	0 (0%)	

6 hours	Cellular outline	5 (100%)	5 (100%)	5 (100%)	0 (0%)	<0.001
	Cytological detail	3 (60%)	5 (100%)	5 (100%)	0 (0%)	
	Nuclear detail	4 (80%)	4 (80%)	5 (100%)	0 (0%)	
	Staining quality	3 (60%)	5 (100%)	5 (100%)	0 (0%)	
	Overall morphology	5 (100%)	5 (100%)	5 (100%)	0 (0%)	

12 hours	Cellular outline	0 (0%)	5 (100%)	5 (100%)	0 (0%)	<0.001
	Cytological detail	0 (0%)	1 (20%)	5 (100%)	0 (0%)	
	Nuclear detail	0 (0%)	2 (40%)	5 (100%)	0 (0%)	
	Staining quality	3 (60%)	4 (80%)	4 (80%)	0 (0%)	
	Overall morphology	0 (0%)	0 (0%)	5 (100%)	0 (0%)	

24 hours	Cellular outline	0 (0%)	0 (0%)	5 (100%)	0 (0%)	<0.001
	Cytological detail	0 (0%)	1 (20%)	5 (100%)	0 (0%)	
	Nuclear detail	0 (0%)	0 (0%)	4 (80%)	0 (0%)	
	Staining quality	0 (0%)	0 (0%)	4 (80%)	0 (0%)	
	Overall morphology	0 (0%)	0 (0%)	5 (100%)	0 (0%)	
